# Correction

**DOI:** 10.1080/27694127.2026.2637293

**Published:** 2026-03-03

**Article title**: TANGO2-related rhabdomyolysis symptoms are associated with abnormal autophagy functioning

**Authors**: Hortense D.C., Sebastian M., Marjolène S., Solène R., Hugo D., Loïc C., Sorana C., Apolline I., Edouard L. G., Anca M., Nicolas G., Laure C., Sylvie F., Arnaud H., Peter V. E., Nicolas D., Julien D., Edor K., & Pascale D. L

**Bibliometrics**: Volume 03, Number 01, pages 2306766

**Journal**: Autophagy Reports

**DOI**: http://dx.doi.org/10.1080/27694127.2024.2306766

This article was originally published without the figure legends, which have now been added, and the article has been republished.

Figure Legends:

Figure 1:

A, B Controls (C) and patients’ (P1, P2 and P3) myoblasts were cultured in Growth Medium (GM) or starved in EBSS for the indicated time points to induce autophagy. A Representative immunoblot showing LC3-II reduction in TANGO2 patients as illustrated in P1. B Quantification of LC3-II at each time point upon starvation in three different TANGO2 patients (P1, P2 and P3). Data are shown relative to beta actin and paired between control and patient on each individual time point. n=3 patients, with at least three technical replicates per patient. *p<0.05, **p<0.01, ***p<0.001 from paired Student t-tests. C Expression of MAP1LC3B and SQSTM1 in basal condition (GM) or upon starvation (EBSS) in patients’ primary myoblasts relative to beta actin and to the controls. Data are pooled per controls and patients and presented as mean + SEM. n=3 controls, n=3 patients with at least two technical replicates per individual. *p<0.05 or enumerated p-values from paired Student t-tests. D-F Control myoblasts were transfected with siRNA oligonucleotide control or against tango2, and four days after transfection, cells were cultured in Growth Medium (GM) or starved in EBSS for the indicated time points. N=4 D Representative immunoblots out of four independent experiments with similar results. E, F TANGO2 (E) and LC3-II (F) protein levels relative to beta actin in siControl and siTANGO2 cells. *p<0.05, or enumerated p-values from paired Student t-tests at each time point. G, H Patients myoblasts’ were transduced with the indicated viruses and stained against FLAG (TANGO2, green) and TOMM20 (mitochondria, red). Scale bar= 20 µm. G Representative images of FLAG and TOMM20 immunostainings. H Quantification of TANGO2 (FLAG) localization to mitochondria (TOMM20) by analysis of Mander’s coefficient of colocalization. *p<0.05 from unpaired Student t-tests.

Figure 2:

A Expression of tango2 zebrafish orthologue at 1 and 2 dpf embryogenic stages in zebrafish as quantified by RT-qPCR. Data are shown as mean + SEM. B Representative pictures of 50 hpf whole zebrafish showing no defect on global morphology. C,D TEER results. (C) Representative swimming trajectories of zebrafish individuals and (D) quantification of average swimming distance, reflecting impaired locomotor abilities of 50 hpf zebrafish from tango2-MOspE3 condition. Each dot represents one embryo. Data are shown as mean +/- SEM. ***p<0,001 and ns non-significant from Kruskal-Wallis and Dunn’s multiple comparisons test. N=5. E Effects of 0,5 and 1 mM atorvastatin (ATV) treatments on zebrafish swimming properties as quantified by average swimming distance parameter by TEER test. Data are shown as mean + SEM. **p<0,01; ***p<0,001; ****p<0,0001 from 2-way ANOVA and Tukey’s multiple comparison test. N=3. F PCR strategy and representative gel electrophoresis of amplification products from 24 and 50 hpf zebrafish showing aberrant splicing products in tango2-MOspE3 zebrafish at both stages. G tango2 mRNA levels relative to beta actin and to controls. p<0,05 and ns non-significant from one way ANOVA and Fisher’s post hoc analysis, N=3. H Monitoring of autophagy flux using the GFP-LC3-RFP-LC3Δ fluorescent probe. Autophagy index as quantified by GFP/RFP ratio by flow cytometry analysis of 30 hpf dissociated zebrafish, showing reduced autophagy flux (increased autophagy index) in tango2-MOspE3 zebrafish cells. Dissociated cells from control-MO zebrafish were treated with 50 nM bafilomycin as a positive control of decreased degradative efficiency of GFP-positive autophagosomes. Data are shown as mean + SEM. **p<0,01 from Kruskal-Wallis and Dunn’s test. N=8. I Schema of transversal sections of 50 hpf zebrafish larvae for immunostainings of the myotomes labelled with myosin heavy chain antibody. J, K Representative images (J) and quantification of average fluorescence intensity (K) from LC3 immunolabeling in myotome sections of 50 hpf zebrafish. Scale bar: 10 µm. *p<0,05 from paired Student t-test. Each pair of data is represented in the same color. K, L Representative images (K) and quantification of average fluorescence intensity (L) from TOMM20 mitochondria immunolabeling in myotome sections of 50 hpf zebrafish. Scale bar: 10 µm. *p<0,05 from paired Student t-test. Each pair of data is represented in the same color. N, O Quantification of mitophagy efficiency in zebrafish 30 hpf larvae. N Representative images of GFP and mCherry signals from the mitoQC probe and of the result of mCherry signal after division by GFP signal (“mCherry/GFP ratio”). O Quantification of mitophagy efficiency. p<0,05 from unpaired Student t-test (n=9).

Figure 3:

A. Average swimming distance from TEER test showing the beneficial effect of 25 mM calpeptin treatment on RM-like features of 50 hpf zebrafish. Data are shown as mean + SEM. *p<0,05; **p<0,01 and ns non-significant from 2-way ANOVA and Tukey’s test. N=4. B. Autophagy index of 30 hpf zebrafish dissociated cells treated with 25 mM calpeptin, showing that calpeptin restores autophagy efficiency of tango2-MOspE3 zebrafish. 50 nM bafilomycin was used as a positive control of decreased degradative efficiency of GFP-positive autophagosomes. Data are shown as mean + SEM. *p<0,05; **p<0,01 from 2-way ANOVA and Tukey’s test. N=6. C Measure of calpains 1/2 activity in 50 hpf zebrafish proteins by fluorescent enzymatic assay (plotted in blue on the left Y axis) and by Western Blot analysis of ATG5 cleavage (plotted in red on the right Y axis). ns non significant and *p<0,05; **p<0,01 ***p<0,001 from 2-way ANOVA followed by Tukey’s multiple comparison analysis for both datasets. D Representative ATG5 immunoblot from a single experiment out of 10 independent experiments with similar results. E, F Western Blot results of controls’ and TANGO2 patients’ primary myoblasts cultured in growth medium (GM) or starved in EBSS, treated or not with 50 mM calpeptin. E Representative LC3 immunoblot from a single experiment out of 9 independent experiments with similar results. F LC3-II protein levels showing that calpeptin treatment improves LC3-II levels upon starvation in control and TANGO2 primary myoblasts. Calpeptin treatment effect was analyzed on GM and starvation conditions. *p<0.05 and ns non-significant, from 2-way ANOVA and Fisher’s post-hoc analysis.

