# Supplementary material for: TANGO2-related rhabdomyolysis symptoms are associated with abnormal autophagy functioning
Source: Autophagy Rep. 2024 Feb 1;3(1):2306766. doi: 10.1080/27694127.2024.2306766 (PMC7617261; doi:10.1080/27694127.2024.2306766)
Supplement: Supplemental Figures_rev.pdf [file KAUO_A_2306766_SM4045.pdf]

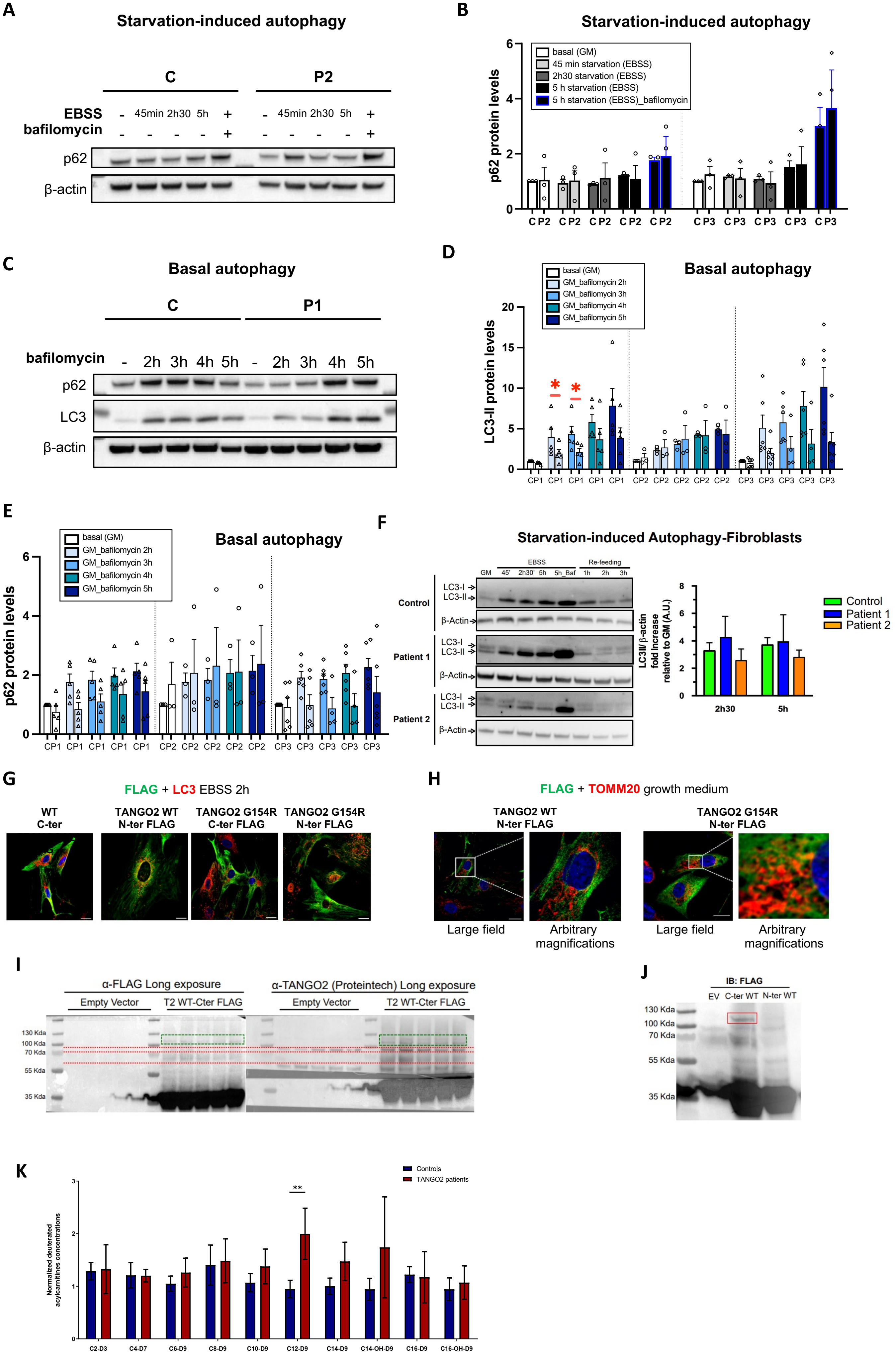

A
Starvation-induced autophagy
C
P2
EBSS
bafilomycin
- 45min 2h30 5h +
p62
β-actin
B
Starvation-induced autophagy
p62 protein levels
basal (GM)
45 min starvation (EBSS)
2h30 starvation (EBSS)
5 h starvation (EBSS)
5 h starvation (EBSS)_bafilomycin
C P2
C P3
C
Basal autophagy
C
P1
bafilomycin
- 2h 3h 4h 5h
p62
LC3
β-actin
D
Basal autophagy
basal (GM)
GM_bafilomycin 2h
GM_bafilomycin 3h
GM_bafilomycin 4h
GM_bafilomycin 5h
LC3-II protein levels
CP1
CP2
CP3
E
Basal autophagy
p62 protein levels
basal (GM)
GM_bafilomycin 2h
GM_bafilomycin 3h
GM_bafilomycin 4h
GM_bafilomycin 5h
CP1
CP2
CP3
F
Starvation-induced Autophagy-Fibroblasts
GM
EBSS
45' 2h30' 5h 5h_Baf
Re-feeding
1h 2h 3h
Control
Patient 1
Patient 2
LC3-I
LC3-II
β-Actin
LC3II/β-actin fold increase relative to GM (A.U.)
Control
Patient 1
Patient 2
2h30
5h
G
FLAG + LC3 EBSS 2h
WT C-ter
TANGO2 WT N-ter FLAG
TANGO2 G154R C-ter FLAG
TANGO2 G154R N-ter FLAG
H
FLAG + TOMM20 growth medium
TANGO2 WT N-ter FLAG
TANGO2 G154R N-ter FLAG
Large field
Arbitrary magnifications
I
α-FLAG Long exposure
α-TANGO2 (Proteintech) Long exposure
Empty Vector
T2 WT-Cter FLAG
130 Kda
100 Kda
70 Kda
55 Kda
35 Kda
J
IB: FLAG
EV
C-ter WT
N-ter WT
130 Kda
100 Kda
70 Kda
55 Kda
35 Kda
K
Controls
TANGO2 patients
Normalized deuterated acylcarnitines concentrations
C2-D3
C4-D7
C6-D9
C8-D9
C10-D9
C12-D9
C14-D9
C14-OH-D9
C16-D9
C16-OH-D9

Supplemental Figure 1

A, B Controls (C) and patients' (P2 and P3) myoblasts were cultured in Growth Medium (GM) or starved in EBSS for the indicated time points to induce autophagy. **A** Representative p62 immunoblot of TANGO2 patient P2. **B** Quantification of p62 protein levels at each time point upon starvation in P2 and P3 TANGO2 patients. Data are shown relative to beta actin and paired between control and patient on each individual time point. n2 patients, with three technical replicates per patient. No statistical difference were obtained from paired Student t-tests. **C-E** Control (C) and patients (P1, P2 and P3) were cultured in Growth Medium without or with bafilomycin for the indicated time points to measure basal autophagy. **C** Representative LC3 and p62 immunoblots of TANGO2 patient P1. **D, E** Quantification of LC3-II protein levels (**D**) and p62 (**E**) at each time point upon starvation in P1, P2 and P3 TANGO2 patients. Data are shown relative to beta actin and paired between control and patient on each individual time point. n3 patients, with at least three three technical replicates per patient. No statistical difference were obtained from paired Student t-tests, excepted when indicated by *p<0,05. **F** Fibroblasts from TANGO2 patients 1 and 2 were cultured as in A Representative LC3 immunoblots and quantification of LC3-II protein levels represented as mean + SEM of N=3 independent experiments after 2h30 and 5h starvation. No statistical difference were obtained from paired Student t-tests. **G** Representative pictures of TANGO2 patients' myoblasts transduced with the indicated viruses, incubated for 2h in EBSS, fixed, and immunostained against FLAG (TANGO2, green) and LC3 (red). Scale bar: 20 µm. **H** Representative pictures of TANGO2 patients' myoblasts transduced with the indicated viruses, fixed, and immunostained against FLAG (TANGO2, green) and TOMM20 (mitochondria, red. Scale bar= 20 µm. **I** Expression of TANGO2-FLAG was evaluated with an anti-Flag antibody (left panel) or Proteintech anti-TANGO2 antibody. The green dashed square highlight bands detected with the two antibodies, indicating their specificity. The red dotted lines highlight the unspecific band characteristic of the Proteintech antibody between 70 and 100 kDa. **J** TANGO2 C-ter WT forms an oligomer between 100-130 kDa. **K** Beta-oxidation flux in whole blood of TANGO2 patients compared to controls. No abnormalities of beta-oxidation flux were observed in patients compared to controls. No difference or **p<0,01 from unpaired Student t-tests.

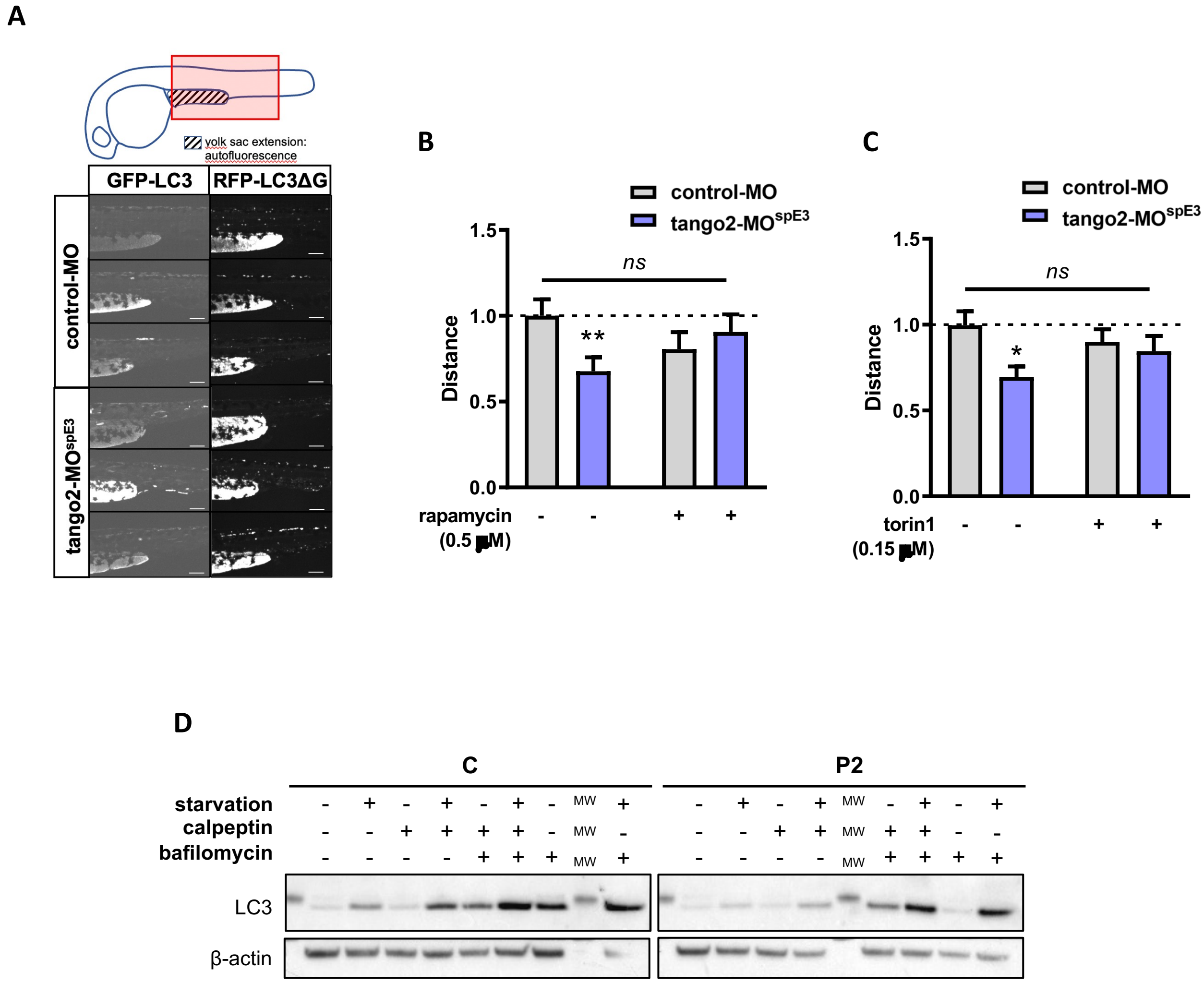

Supplemental Figure 2

A Representative images of 30 hpf alive zebrafish expressing the GFP-LC3-RFP-LC3Δ fluorescent probe. Scale bar 100 µM. **B, C** Average swimming distance from TEER test showing the beneficial effect of 0,5 µM rapamycin (**B**) and 0,15 µM torin 1 (**C**) treatment on RM-like features of 50 hpf zebrafish. Data are shown as mean + SEM. *p<0,05; **p<0,01 t from 2-way ANOVA and Tukey's test. N=4. **D** LC3 immunoblots showing the benefical effect of calpeptin treatment on LC3-II levels in TANGO2 primary myoblasts as demonstrated by increased levels of LC3-II in the presence of bafilomycin.
